# Sensori-motor adaptation to knee osteoarthritis during stepping-down before and after total knee replacement

**DOI:** 10.1186/1471-2474-6-21

**Published:** 2005-04-26

**Authors:** L Mouchnino, N Gueguen, C Blanchard, C Boulay, G Gimet, J-M Viton, J-P Franceschi, A Delarque

**Affiliations:** 1 Laboratory of Movement and Perception, Faculty of Sport Sciences, 163 av. de Luminy 13288 Marseille cedex 9, France; 2 Department of Physical Medicine and Rehabilitation, Université de la Méditerranée, 92 rue A. Blanqui 13005 Marseille, France; 3 Department of Orthopedic Surgery, CHU Conception, bd. Baille, 13005 Marseille, France

## Abstract

**Background:**

Stepping-down is preceded by a shift of the center of mass towards the supporting side and forward. The ability to control both balance and lower limb movement was investigated in knee osteoarthritis patients before and after surgery. It was hypothesized that pain rather than knee joint mobility affects the coordination between balance and movement control.

**Methods:**

The experiment was performed with 25 adult individuals. Eleven were osteoarthritic patients with damage restricted to one lower limb (8 right leg and 3 left leg). Subjects were recruited within two weeks before total knee replacement by the same orthopedic surgeon using the same prosthesis and technics of surgery. Osteoarthritic patients were tested before total knee replacement (pre-surgery session) and then, 9 of the 11 patients were tested one year after the surgery when re-educative training was completed (post-surgery session). 14 adult individuals (men: n = 7 and women: n = 7) were tested as the control group.

**Results:**

The way in which the center of mass shift forward and toward the supporting side is initiated (timing and amplitude) did not vary within patients before and after surgery. In addition knee joint range of motion of the leading leg remained close to normal before and after surgery. However, the relative timing between both postural and movement phases was modified for the osteoarthritis supporting leg (unusual strategy for stepping-down) before surgery. The "coordinated" control of balance and movement turned to be a "sequential" mode of control; once the body weight transfer has been completed, the movement onset is triggered. This strategy could be aimed at shortening the duration-time supporting on the painful limb. However no such compensatory response was observed.

**Conclusion:**

The change in the strategy used when supporting on the arthritis and painful limb could result from the action of nociceptors that lead to increased proprioceptor thresholds, thus gating the proprioceptive inputs that may be the critical afferents in controlling the timing of the coordination between balance and movement initiation control.

## Background

Anticipatory postural adjustments (APAs) precede voluntary lower limb movements, as shown by experiments in which the limb to be moved initially supported the body weight (leg flexion [1,2]; lateral leg raising [3]; gait initiation [4]). In these cases, movement is preceded by a shift of the center of mass (CM) towards the supporting side and forward as in gait initiation. This anticipatory CM shift, aimed at unloading the leg to be moved and creating the condition for progression, is initiated by the generation of forces (thrust exerted on the ground) that shift the CM. Although no specific receptors exist that detect CM position and shift, it can be indirectly estimated through measurements of the center of pressure (CP). Morasso and Schieppati [5] showed the acceleration of the CM correlates highly with the CM-CP difference, as a consequence of physical laws. It has been hypothesized that pressure afferent inputs play a major role in determining the actual position of body mass to be balanced over the feet (i.e. the CM).

Load-detecting and position-sense afferents might be candidates for monitoring balance regulation, as shown by Dietz et al. [6], Eklund [7] and Roll and Roll [8] for proprioceptive information and by Magnusson et al. [9] and more recently by Kavounoudias et al. [10] for plantar cues. Afferent inputs also have phase-dependent effects during gait (see [11] for review). Other afferents such as noxious sensory afferents can influence balance control and could have deteriorating effects on postural control mechanisms [12]. Nociceptive primary afferent fibers have a peripheral action by way of dorsal root reflexes. Rossi et al. [13] showed in the case of foot pain that proprioceptive activity is profoundly influenced by nociceptive reflex action, indicating how closely the two functions of the two systems may be associated during natural movements.

We investigated the ability to control both balance and lower limb movement initiation in knee osteoarthritis patients, in a stepping down task. Stepping down requires a transition from a bipedal to a monopedal stance (as in the leg raising task) in addition with a forward propulsion prior to heel-off (as in gait initiation). These APAs precede any movement of the leading lower leg. In knee osteoarthritis patients, knee joint mobility is impaired, as are the torques exerted by this joint when supporting the body weight. Knee joint excursion and muscle strength are intrinsic elements of stiffness and are both affected by knee osteoarthritis. Among these physical decay mechanisms, the main factor is pain of both inflammatory and mechanical origins. The inflammatory pain results from the effects of a variety of endogenous chemical agents released from damaged cells at the knee joint level [13] and entering the damaged region (i.e. at the thigh level).

It is hypothesized that pain rather than knee joint mobility and muscle strength could change the coordination between APAs and movement initiation leading to compensatory mechanisms in osteoarthritis patients.

## Methods

### Subjects

The experiment was performed with 25 adult individuals. Eleven were osteoarthritic patients (mean age: 69 years from 45 to 82, men: n = 5 and women: n = 6; mean weight: 75 kg from 63 to 109; mean height: 1.64 m from 1.50 to 1.87) with damage restricted to one lower limb (unilateral symptomatic knee arthritis; 8 right leg and 3 left leg). Subjects were recruited within two weeks before total knee replacement by the same orthopedic surgeon using the same type of posterior cruciate-sparing prosthesis and technics of surgery were used in all patients.

A control group of 14 healthy adults were tested in this experiment (men: n = 7 and women: n = 7) (mean age: 72 years old from 66 to 81; mean weight: 65 kg from 47 to 85; mean height: 1.66 m from 1.50 to 1.82).

### Protocol

Subjects were instructed to step down from a platform with a standard step height of 170 mm. Twenty randomized trials (ten with each leg) per subject were performed. Light Emitting Diodes located to the left and to the right signaled to the subject when to start their movement and which leg they had to move first.

**Figure 1:**
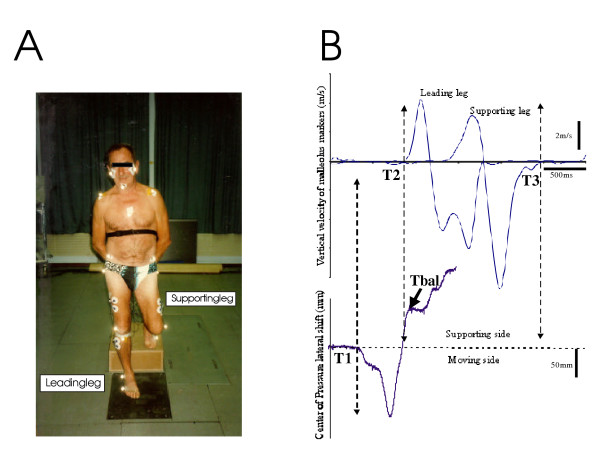
Phases of the motor act in stepping down movement (A). Reference times were measured from 2 curves (B). *Top*, The reference times plotted on the vertical velocity curve of the malleolus marker of the leading leg (T2) and of the supporting leg (T3) correspond respectively to the onset and offset of the movement phase. *Bottom*: lateral CP curve (T1) corresponds to the onset of CP change and Tbal to the end of the ballistic CP shift.

A wooden platform was positioned on a force platform and in relation to the edge of a second force platform (Fig. 1A) so that each subject could comfortably land in the middle of this second force plate. Subjects kept the landing platform in view with peripheral vision and aimed central vision forward to a go signal given by the experimenter.

A trial consisted of a subject stepping off the first platform with either the right or the left foot and landing with the "leading" foot in the middle of the force-plate. Touch down with the leading foot was followed by stepping off the platform and over the landing force plate with the "supporting" foot.

Osteoarthritic patients were tested before total knee replacement (pre-surgery session) and then, 9 of the 11 patients were tested one year after the surgery when re-educative training was completed (post-surgery session). The control group was tested during one session. For safety, a researcher and medical doctor stood behind and laterally to each patient while stepping, but all subjects were able to execute the task without assistance.

### Data collection

The kinematic analysis was performed by an automatic TV-image processor (EL.I.TE. system). TV cameras worked at a sampling frequency of 100 Hz, while system accuracy was 1 part in 3000. Under the present experimental condition, the field of view explored was 2*2*3.5 m and the accuracy was less than 1 mm.

Sixteen light-reflecting markers were placed on anatomical landmarks: bilaterally, on the external edge of the orbits, the acromions, the anterior-superior iliac spines, the greater trochanters, the external part of the lateral femoral condyles, the anterior tibial tuberosities, the lateral malleoli, and the 5th metatarsal heads. In this study we did not use the markers placed on the orbits, on the acromions, and on the 5th metatarsal heads. (Figure 1A). Two cameras were placed 3.5 m in front of the subject.

Electromyographic recordings (EMG) were made from 2 muscles Vastus lateralis (VL, knee extensor) on both sides of the subject, by means of bipolar surface electrodes spaced 2 cm apart. Preamplifiers were placed next to the recording electrodes. The EMG signals were amplified (gain of 1000), band-pass filtered (10 Hz to 200 Hz), digitized at 500 Hz, and rectified.

The ground reaction forces were recorded at a frequency of 500 Hz with the subjects standing on an AMTI force platform and landing on a Kistler force platform.

### Data analyses

The onset of the lateral shift of the center of pressure (T1) was taken as the onset of the APAs (Fig 1B). The **postural phase **started with the first CP change (T1) measured with the force platform and ended with the end of the ballistic CP shift towards the supporting side (Tbal), which corresponds to a breakdown in the M/L CP curve (Fig. 1B).

The vertical velocity profile at the ankle (malleolus marker) was approximately bell-shaped, with a single maximum. The onset of leg flexion (T2) was taken as the end of the initial period of zero vertical velocity (+/-5% of the maximal velocity) of the leading leg (malleolus marker). The end point (T3) was defined as the position at which the leg that will be the supporting one returned back to zero velocity after the movement (Fig. 1B). T2–T3 defined **the movement phase**.

EMG analysis was performed by calculating **latencies and areas of integrated EMG **activities or bursts. The resting activities were measured in each trial in the 300 ms of recording preceding the signal onset in order to determine the background EMG activity. The mean and standard deviation of this background activity were then calculated for each subject. Timing and intensity measurements were performed. For the timing measurement, the onset and the end of EMG bursts were defined as the times when the EMG activity increased above or decreased below a threshold level set at two times the standard deviation of the background activity. The duration of the burst was also calculated. The intensity of muscular activity was calculated by subtracting the baseline from the EMG activity level reached 300 ms after the activity had increased above the threshold level. In the analysis of the changes in muscle activity profiles, the first step involved two phases: the pre-activation phase lasting 300 ms prior to the landing (first signal recorded by the landing platform), and the activation phase computed during 300 ms following the ground contact.

Next, the EMG activity was windowed before ground contact (**pre-activation**: from -300 ms to -150 ms and -150 ms to ground contact) and after the ground contact (**activation**: from the ground contact to 150 ms and 150 to 300 ms).

### Statistical analysis

To determine modifications caused by the surgery, dependent variables were tested in a first step, using a 2*2 (pre-, post-surgery * sound, arthritis leg) repeated measures ANOVA.

In a second step, comparison with a control group was done to document that, post-surgery, behavior of patients was not different from that of a control group. Dependent variables were assessed using a 2*2 (post-surgery patients, control group * 2 sides) between subjects ANOVA. The Newman-Keuls post-hoc test was used to assess the difference between factors. The level of significance was set at 5%.

## Results

### Pain intensity and passive joint mobility assessments

The average pre-surgery Hospital for Special Surgery (HSS) score was 59.1 (+/- 10.15) with a maximum of 100. The mean pain Visual Analog Scale (VAS) value was 49 mm (+/- 9) (VAS; worst pain ever 100 mm, no pain 0 mm). The mean post-surgery HSS score was 80.8 (+/- 8.4) and the VAS value was 7 (+/- 9). The VAS value vas obtained just after stepping-down task. The passive mobility of the knee joint was tested for all patients. The average pre- and post-surgery mobilities were 113 degrees (+/- 21) and 105° (+/- 18) respectively, whereas the mobility of the sound knee was 128° (+/- 11).

### Anticipatory Postural Adjustments

The osteoarthritis patients exhibited an increase of the duration of the postural phase (T1-Tbal; Fig. 1B) after surgery. This effect was however not statistically significant [F(1,4) = 5,67; p = 0.075]. On average, the total duration of the postural phase was longer (835 ms +/- 207) in the post-surgery session, than in the pre-surgery session (652 ms +/-143) and no side-effect was observed within patients. Post-surgery patients did not recover a duration similar (p < 0.001) to that observed in the control group (543 ms +/- 107).

**Figure 2:**
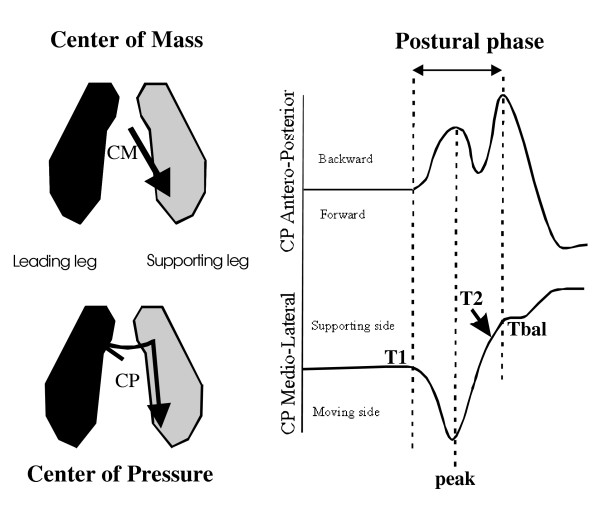
Schema of the horizontal shift of the center of mass (CM) and associated center of pressure (CP) (*left part*) and description of the M/L and A/P CP curves (*right part*). The dotted lines show the time-relationships between each component. Note that the M/L thrust (T1-Peak) coincides with the first backward CP shift, and that during the unloading component of the M/L CP shift, the second backward shift occurs, which corresponds to heel off (T2).

By contrast, the onset of this phase in terms of "thrust" exerted onto the ground (Fig. 2) was not different in patients before and after surgery [F(1,4) = 0.038; P = 0.85]. The A/P and M/L peaks remained synchronized before (22 ms+/-60) and after surgery (16 ms +/- 17). These events were tightly coupled in patients after surgery as in the control group (1 ms +/- 26). In addition, the M/L peak amplitude was not different in patients between pre- (270 mm +/-45) and post-surgery sessions (257 mm +/-37). After surgery, the M/L thrust was close to that observed in the control group (258 mm +/-35; p = 0.88).

### Movement performance

The total duration of the movement phase (T2–T3) was not different in patients [F(1,4) = 1.80; p = 0.24] (Pre-surgery: 1694 ms +/- 355 ; Post-surgery 1502 ms +/- 230). The movement duration in post-surgery was similar (p = 0.066) to that observed in the control group (1402 ms +/- 193).

The maximal flexion reached by the leading leg did not differ statistically in patients before and after surgery [F(1,6) = 5.44; p = 0.058] (see table. 1) and no side-effect was observed within patients. After surgery, the maximal flexion was close to that observed in the control group (p = 0.37). By contrast, considering the flexion of the leg that was previously the supporting leg, after surgery, patients decreased the leg flexion [F(1,5) = 19,8; p = 0.006] (see table. 1) and no side-effect was observed. However, in post-surgery session, the maximal flexion of the supporting leg remained reduced compared to the control group (p = 0.0017).

**Table 1:** Maximal knee joint angle reached during the stepping-down performance of the leading leg and of the supporting leg during the swing phase.

**Maximal knee joint angle**			
***Leading leg***	*Controls*	*Patients before surgery*	*Patients after surgery*

Right / Sound	45.4° +/-4.7	49.2° +/-10.5	58.3° +/-23.4
Left / arthritis / operated	46.2° +/-7.3	33.7° +/-14	40.9° +/-12

			

***Supporting leg***	*Controls*	*Patients before surgery*	*Patients after surgery*

Right / Sound	79.9° +/-10	81.2° +/-6.3	67.3° +/-29.1
Left / arthritis / operated	82.7° +/-4.4	55.3° +/-14	52° +/-21.2

### Time-relationships between APAs and movement initiation

The stepping down movement of the leading leg was triggered while the unloading phase (peak-Tbal, Fig. 2) was being performed, before the M/L CP shift was completed. The time-relationships between unloading (Tbal) and stepping down initiation (T2) differed in patients before and after surgery [F(1,4) = 15.53; p = 0.016]. Before surgery, in patients who used the arthritis limb as the supporting limb (unusual strategy), the movement initiation was delayed and coincided (-64 ms +/-452) with the end of the lateral unloading. This result, however, varied widely, as shown by the high standard deviation. Post-surgery, stepping down is triggered largely before the unloading is completed (sound supporting leg: -514 ms +/-60; operated supporting leg: -492 ms +/-176). Post-surgery patients did not recover an anticipation similar to that observed in the control group (-214 ms +/-40) (p < 0.001).

The delayed movement initiation (T2) when supporting on the arthritis leg before surgery, might be aimed at shortening the duration of the supporting phase for the painful leg. This was not the case, however, because there was no clear side-effect [F(1,4) = 6.33; p = 0.065] on the duration of the monopodal stance. In addition, this duration was even longer [F(1,4) = 19.8; p = 0.011] before than after surgery (797 ms+/-197 and 681 ms+/-156, respectively). The post-surgery duration decreased to a value close (p = 0.38) to that observed in the control group (644 ms +/-49).

### Weight acceptance

**Figure 3:**
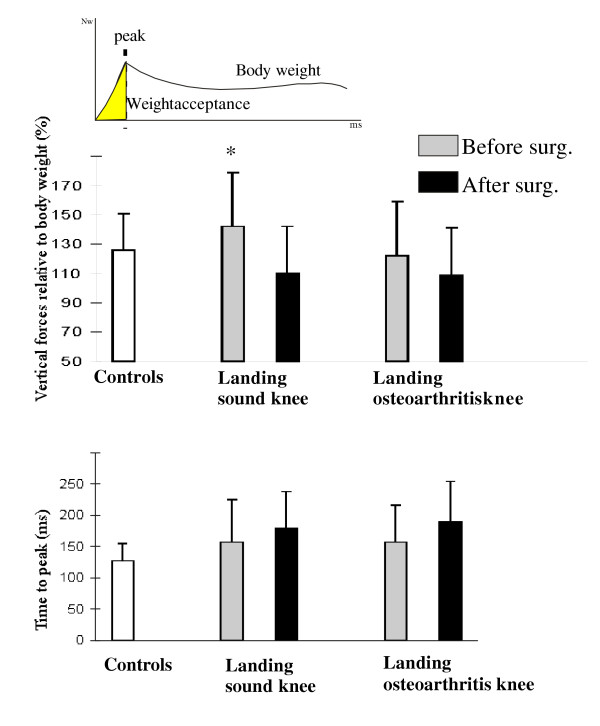
Schema of the vertical ground reaction force recorded on the landing force platform. Weight acceptance was from the ground contact to the peak and was calculated in percentage relative to the body weight to normalize the data for all the subjects.

The adaptation of the weight acceptance is illustrated in Fig. 3. The ground impact, defined as the maximal value of the vertical ground reaction force and normalized to the body weight, did not differ in patients before and after surgery [F(1,4) = 3.37; p = 0.14]. However, in patients landing on the sound leg (i.e. using the arthritis leg as the supporting leg) before surgery, the ground impact increased (142 % +/-36) [side-effect F(1,4) = 7.59; p = 0.05] compared to those landing on the arthritis leg (118 %+/-37) (Fig. 3). This result indicated a reduced breaking capacity of the supporting knee joint during the monopodal stance, which enhanced the forthcoming ground impact. After surgery, the ground impact decreased to a value close to that observed in the control group (p = 0.78) (Fig. 3).

There was no significant difference in "time to peak" of the vertical force (weight acceptance velocity) for both sides in patients before and after surgery [F(1,4) = 3.37; p = 0.14] (Fig 3).

### EMG activities associated with ground contact

**Figure 4:**
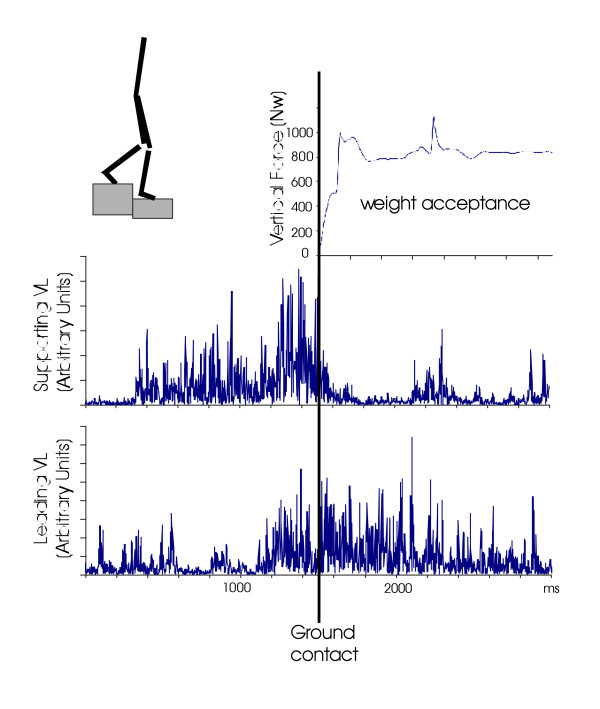
Kinetic and rectified EMG patterns recording with one control subject. The EMGs were recorded at a proximal level (VL, Vastus lateralis) for both sides. Note the supporting and leading VL activity prior to the ground contact.

The comparison between kinetic events and associated EMG pattern points out some differences. First, during the swing phase, the moving limb exhibited a pre-activation of the VL before the ground contact. The leading VL pre-activation was correlated with increasing activity of the VL on the supporting side (Fig. 4). The onset of the pre-activation of the VL muscle of the leading limb did not differ in patients before and after surgery [F(1,5) = 1.63; p = 0.25]. However, in pre-surgery session, the pre-activation occurred earlier [side-effect F(1,5) = 11.84; p = 0.018] when landing on the arthritis leg (-414 ms+/-90) than when landing on the sound leg (-335 ms +/-90). This was also observed for the post-surgery sessions (345 ms +/-67 and -298 ms +/-74, respectively).

**Figure 5:**
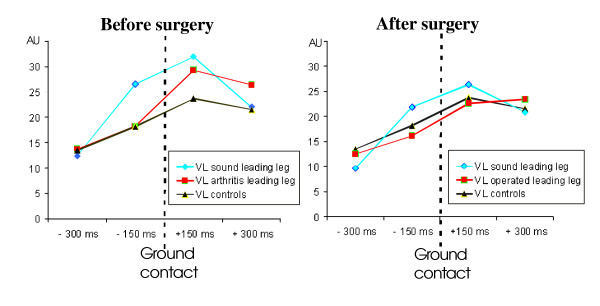
Dynamic profiles of VL activation recorded on the forthcoming landing leg before and after surgery. EMG data are windowed each 150 ms from 300 ms before ground contact to 300 ms after ground contact (Arbitrary Units, AU).

VL muscle activation was not statistically different in patients before and after surgery [F(1,5) = 0.5; p = 0.50] (Fig. 5). The pre-activation increased [window-effect F(3,15) = 14.36; p < 0.001] from the first window (-300 ms to -150 ms) to the second (-150 ms to ground contact) and to the third (ground contact to 150 ms). However, when landing on the sound leg, the activity of the leading VL strongly increased before the ground contact (-150 ms to ground contact) [interaction side*window [F(3,15) = 5.13; p = 0.012]. Note that in this latter case, the VL of the leg to be stepped down supported 140% of the body weight. No such increase was observed in patients landing on the arthritis leg. Post-surgery (Fig. 5), this enhanced activity no longer exhibited differences compared to the control group (P = 0.39).

## Discussion

The present study examined whether the coordination between balance and movement remained unchanged in patients as in controls. It was hypothesized that pain rather than knee joint mobility could change the coordination between balance and movement leading to compensatory mechanisms.

### Lack of changes in the APAs

It was found that the way the Center of Mass shifts towards the supporting side and forward is initiated during the postural phase (T1-Tbal) did not changed in patients. The same observation was made for controls. These results are in agreement with that characterizing patients before surgery reported during lateral stepping by Viton et al. [15]. The proactive mechanism controlling the APAs may be triggered in absence of peripheral feedback as proposed by Forget and Lamarre [16,17] for a forearm unloading (waiter task) performed by a patient without proprioception. Bent et al. [18] reported before voluntary forward step performed without vision, that the initiation phase is run in a feedforward manner without vestibular influence. In the present experiment the presence of pain, mobility and muscle strength possible changes were not sufficient to influence the initial control of the APAs.

### The effects of arthritis supporting leg on triggering the leg movement

The coordination between balance and movement initiation (T2) is changed before surgery : movement onset was delayed until the end of the postural phase when patients used an unusual strategy to support themselves on the arthritis limb. The previous coordinated control of balance and movement (i.e. movement onset is initiated while the unloading of the leading leg is being performed) changed to a sequential mode of control; once the CM lateral shift was completed, the movement onset then was triggered. This change in the coordination leads to a forward fall as shown by the ground impact enhancement associated with an increased amount of muscle activity just prior to ground contact. There are several interpretations to explain the sequential strategy.

One possible explanation would have been that the stepping down movement is delayed to decrease the duration of the monopodal stance phase on the painful limb. However, no such compensatory mechanism was observed as shown by the longer duration of the monopodal stance on the arthritis leg as compared to the sound supporting leg.

The second interpretation of the time lag between postural adjustments and movement initiation observed before surgery might result from the lack of knee joint mobility. This, however, is not shown by the knee joint maximal flexion during stepping down which is even greater in pre-surgery session than in post-surgery. The breaking ability of the supporting arthritis leg (absorption by the knee extensor) could not intervene because at that time both feet are on the ground.

A third possibility could be related to an adapted motor command due to fear of pain. During the pre-surgery session, the patients were asked whether they have pain in quiet standing (i.e. before stepping down). None of the subjects reported having perceived any pain at that time. However, they reported to suffer from the arthritis limb when exerting pressure. In that case, a less painful strategy might be to avoid pressure on the arthritis leg in decreasing the amplitude of the M/L thrust. No such compensatory response was observed. In addition, twenty trials per subjects were collected in the pre- as in the post-surgery sessions. If fear of pain is the main contributing reason to observed changes in the coordination, the thrust amplitude would decrease from the first to the following trials. No such decrease was observed.

The sequential organization between postural phase and movement performance together with the high variability of the timing accounted to a reduced accuracy in the integration of sensory information. Pain modulation of movement might happen in many ways. One of this mechanism may involved presynaptic inhibition [19] produced by nociceptive action. Presynaptic control of Ia afferents from extensor muscles may shape the amplitude, duration, and timing of the stance phase of locomotion [20]. Acute arthritis and associated nociceptive stimulation might lead to increased proprioceptor thresholds thus gating the proprioceptive inputs, as it was previously reported by Rossi et al. [13] during locomotion in the case of foot pain. After surgery, pain is removed and this coordination becomes normal in addition with a slowing strategy of the body weight transfer. These observations emphasize the deteriorating effects of a nociceptive stimulation in controlling the timing of the coordination between balance and movement initiation control. This timing is normally controlled by the afferents of proprioceptive origin (see for review [21]). In that case, the load feedback mechanisms play a crucial role in phase-switching during the leg movement task, as reported for the 1b activity of ankle extensors in the switch-phase of locomotion [11]. This could certainly lead to the hypothesis of a combined effect of nociceptive and proprioceptive afferents in the posture-movement coordination as it was reported by Blouin et al. [12] for balance control.

## Conclusion

To conclude, pain more than fear of pain or knee joint mobility and muscle strength appears to be the relevant factor that disturbed the coordinated control between balance and movement. After surgery no more pain is noticeable and the motor patterns were restored to as close to "normal" as possible.

## Competing interests

The author(s) declare that they have no competing interests.

## Authors' contributions

LM, NG, JMV and AD participated in the conception and design of the study. LM, NG, CBl Cbo and GG performed data analyses. JPF was the orthopedic surgeon. All authors read and approved the final manuscript.

## Pre-publication history

The pre-publication history for this paper can be accessed here:


